# A radial basis classifier for the automatic detection of aspiration in children with dysphagia

**DOI:** 10.1186/1743-0003-3-14

**Published:** 2006-07-17

**Authors:** Joon Lee, Stefanie Blain, Mike Casas, Dave Kenny, Glenn Berall, Tom Chau

**Affiliations:** 1 Bloorview Kids Rehab, Toronto, Ontario, Canada; 2 Institute of Biomaterials and Biomedical Engineering, University of Toronto, Toronto, Ontario, Canada; 3 The Edward S. Rogers Sr. Department of Electrical and Computer Engineering, University of Toronto, Toronto, Ontario, Canada; 4 The Hospital for Sick Children, Toronto, Ontario, Canada; 5 North York General Hospital, Toronto, Ontario, Canada

## Abstract

**Background:**

Silent aspiration or the inhalation of foodstuffs without overt physiological signs presents a serious health issue for children with dysphagia. To date, there are no reliable means of detecting aspiration in the home or community. An assistive technology that performs in these environments could inform caregivers of adverse events and potentially reduce the morbidity and anxiety of the feeding experience for the child and caregiver, respectively. This paper proposes a classifier for automatic classification of aspiration and swallow vibration signals non-invasively recorded on the neck of children with dysphagia.

**Methods:**

Vibration signals associated with safe swallows and aspirations, both identified via videofluoroscopy, were collected from over 100 children with neurologically-based dysphagia using a single-axis accelerometer. Five potentially discriminatory mathematical features were extracted from the accelerometry signals. All possible combinations of the five features were investigated in the design of radial basis function classifiers. Performance of different classifiers was compared and the best feature sets were identified.

**Results:**

Optimal feature combinations for two, three and four features resulted in statistically comparable adjusted accuracies with a radial basis classifier. In particular, the feature pairing of dispersion ratio and normality achieved an adjusted accuracy of 79.8 ± 7.3%, a sensitivity of 79.4 ± 11.7% and specificity of 80.3 ± 12.8% for aspiration detection. Addition of a third feature, namely energy, increased adjusted accuracy to 81.3 ± 8.5% but the change was not statistically significant. A closer look at normality and dispersion ratio features suggest leptokurticity and the frequency and magnitude of atypical values as distinguishing characteristics between swallows and aspirations. The achieved accuracies are 30% higher than those reported for bedside cervical auscultation.

**Conclusion:**

The proposed aspiration classification algorithm provides promising accuracy for aspiration detection in children. The classifier is conducive to hardware implementation as a non-invasive, portable "aspirometer". Future research should focus on further enhancement of accuracy rates by considering other signal features, classifier methods, or an augmented variety of training samples. The present study is an important first step towards the eventual development of wearable intelligent intervention systems for the diagnosis and management of aspiration.

## Background

### Dysphagia and aspiration

Dysphagia generally refers to any swallowing disorder. Impaired swallowing may result from mechanical disorders due, for example, to the removal or reconstruction of swallowing structures secondary to surgery for cancer [1] or anatomic abnormalities of the mouth, nose, pharynx, larynx, trachea and esophagus [2]. Compromised swallowing function can also be neurological in origin. Examples include lesions in the brain stem or peripheral cranial neuropathies [3] and cortical lesions [4]. Disorders of deglutition are common in neurological impairments due to stroke, cerebral palsy or acquired brain injury. Children with dysphagia often have heightened risk of aspiration.

Aspiration is entry of foreign material into the airway below the true vocal cords [5] accompanied by inspiration [6]. Approximately 25% of individuals at risk of aspiration do so in a "silent" manner [7], with no overt physiological signs (e.g. coughing, face turning red, uncoordinated breathing) and care-givers may have no warning that an aspiration has occurred.

### Magnitude of problem

Dysphagia afflicts an estimated 15 million people in the United States [8]. The incidence of dysphagia is particularly significant in acute care settings (25–45%) and long-term care units (50%) [9]. In the United States, approximately 50,000 persons die annually from aspiration pneumonia [10].

Silent aspiration is especially prominent in children with dysphagia, occurring in an estimated 94% of that population [11]. The occurrence of diffuse aspiration bronchiolitis in children with dysphagia is generally widespread [12]. The increased risk of aspiration bears serious health consequences such as dehydration, malnutrition, chronic lung disease and acute aspiration pneumonia [2,11]. The latter is an expensive outcome that often requires extended hospitalization. Pulmonary aspiration can also evolve to include systemic complications such as bacteremia, sepsis, and end-organ consequences of hypoxia and death [13]. Chronic aspiration is therefore an insidious problem that tremendously diminishes quality of life, not only compromising a child's physical, but social, emotional and psychosocial well-being.

### Current aspiration detection methodologies

Only the most prevalent methods of aspiration detection in the current literature are reviewed. The modified barium swallow using videofluoroscopy is the current gold standard for diagnosis of aspiration [14]. Its clinical utility in dysphagia management continues to be asserted (e.g., [15,16]). The patient ingests barium-coated material and a video sequence of radiographic images is obtained via X-radiation. The modified barium swallow procedure is costly both in terms of time and labor (approximately 1,000 health care dollars per procedure in Canada), and renders the patient susceptible to the nonstochastic effects of radiation [17].

Fibreoptic endoscopy, an invasive technique in which a flexible endoscope is inserted transnasally into the laryngopharynx, has also been widely applied, for example, in the diagnosis of post-operative aspiration [18] and bedside identification of silent aspiration [19]. Fibreoptic endoscopy is generally comparable to the modified barium swallow in terms of sensitivity and specificity for aspiration identification (e.g., [20,21]), with the advantage of possible bedside assessment.

Pulse oximetry has also been proposed as a non-invasive adjunct to bedside assessment of aspiration risk (e.g., [22,23]). However, several controlled studies comparing pulse oximetric data to videofluoroscopic [24] and fibreoptic endoscopic evaluation [25,26] have raised doubts about the existence of a relationship between arterial oxygen saturation and the occurrence of aspiration.

Cervical auscultation involves listening to the breath sounds near the larynx by way of a laryngeal microphone, stethoscope or accelerometer [27] placed on the neck. It is generally recognized as a limited but valuable tool for aspiration detection and dysphagia assessment in long-term care [27-29]. However, when considered against the gold standard of videofluoroscopy, bedside evaluation with cervical auscultation yields limited accuracy in detecting aspirations [27] and abnormalities of swallowing [30]. Indeed, our recent research shows that aspirations identified by the clinician, represent only 45% of all aspiration sounds [6].

Swallowing accelerometry [31] is closely related to cervical auscultation, but has entailed digital signal processing and artificial intelligence as discrimination tools, rather than the trained clinical ear. In clinical studies, accelerometry has demonstrated moderate agreement with videofluoroscopy in identifying aspiration risk [32] while the signal magnitude has been linked to the extent of laryngeal elevation [31]. Fuzzy committee neural networks have demonstrated extremely high accuracy at classifying normal and "dysphagic" swallows [33].

Administration of existing procedures, such as videofluoroscopy or fibreoptic endoscopy, usually requires expensive equipment and specially trained professionals such as a speech-language pathologist, radiologist or otolaryngologist [34]. Further, the invasive nature of procedures such as fibreoptic endoscopy does not bode well with children and therefore the method cannot be practically administered for extended periods of feeding. Clearly, there is an identified but unmet need for an economical [22], non-invasive and portable method of paediatric aspiration detection [32], at the bedside [25] and outside of the institutional setting.

As an important step towards addressing this unmet need, we present details of a classifier for automatic detection of aspiration in children with dysphagia. In the next section, we outline the methods pursued in developing the classifier. Subsequently, we report quantitative classification results using different candidate feature sets. We also briefly describe one possible hardware implementation of the classifier. The paper closes with a discussion of the merits and limitations of the classification algorithm and future directions of research. It is anticipated that such a classifier once implemented in a portable computing platform could assist caregivers in their interventions to manage heightened aspiration risk.

## Methods

### Representation of swallowing activity

Based on the clinical appeal of cervical auscultation and the recent success of swallowing accelerometry described above, we decided to represent swallowing activity, in particular, aspirations and safe swallows, by way of anterior-posterior vibrations at the neck. This choice of representation proved meaningful in our previous study of pediatric aspirations [6].

### Data collection for system design and evaluation

In order to construct an automatic classification method, we required examples of aspiration and swallow vibrations. To this end, one hundred and seventeen children suspected to be at risk of aspiration were recruited to this study. Parents or caregivers gave their informed consent prior to each child's participation. The protocol was approved by the Research Ethics Board of Bloorview Kids Rehab (Canada). The mean age of the participants was 6.0 ± 3.9 years with 64 males and 53 females. Swallowing difficulty in all the participants was neurological in origin, with the overwhelming majority having a primary diagnosis of cerebral palsy.

**Figure 1:**
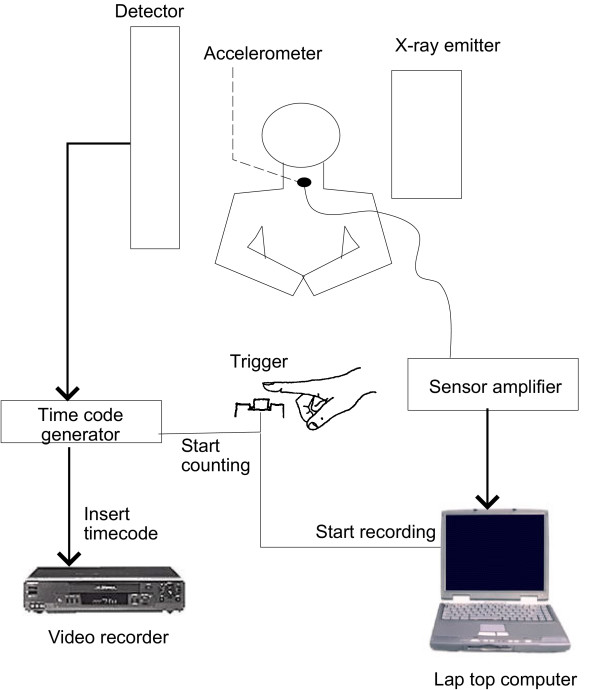
Data collection set-up for the simultaneous acquisition of time-synchronized videofluoroscopic and accelerometric data.

Lateral fluoroscopic video (General Electric X-ray System, RFX-90) of the cervical region and simultaneous, time-synchronized accelerometric data were collected from each child during routine videofluoroscopic examination. As shown in Figure 1, a small single-axis accelerometer (EMT 25-C, Siemens) was attached to the child by way of double-sided tape, infero-anterior to the thyroid notch. This accelerometer, with a sensitivity of 80 mV/g, was chosen for its flat frequency response, from 30 Hz to 20 kHz, covering the previously reported range of frequencies relevant to swallowing activities [35,36]. The accelerometer signal was sampled at 10 kHz. The child was fed a barium-coated bolus of varying consistencies as per the modified barium swallow procedure [15]. Categories of consistencies included thick, medium and thin purées, honey, nectar, thin liquid and soup. Video X-rays were recorded on tape in analog form (Panasonic VCR, model AG-6200), while accompanying time-synchronized vibration signals were amplified and recorded onto a laptop computer (Apple PowerBook G3, 266 MHz) via an external 12-bit data acquisition unit (Biopac, model MP100). The raw data were denoised by wavelet soft-thresholding using a Daubechies-4 filter. Video X-ray recording was triggered by the initial activation of the X-ray emitter, operated by the presiding radiologist. Time-stamping of the video (FORA video timer, model VTG-55) and recording of the vibration signal were triggered simultaneously, by the presiding pediatrician via a pushbutton switch, upon observation of swallow initiation. In this manner, the time code on the analog video corresponded to the time index of the digital recording of the vibration signal.

The video records were subjected to retrospective blind review by a committee of three to four clinical experts, for the purpose of aspiration identification. The vibration signals associated with the identified instances of aspirations were carefully extracted, reviewed by committee and checked for sound quality. Each aspiration sample was further assigned one of four possible descriptive labels based on a consensus classification of the sound by the committee of the clinical experts. These labels are summarized in Table 1. Additional details of aspiration signal extraction can be found in [6]. By this procedure, 94 aspiration and 100 swallow signals were extracted.

**Table 1:** Descriptive labels of aspiration signals

**Label**	**Outstanding quality in signal**
squeak	Characteristic high frequency inspiratory squeak
crunch	Dull crunching sound
click	Short single click
clip	High amplitude sound with fuzzy quality

### Feature extraction

Critical to any successful classifier is the prudent extraction and selection of discriminatory features. Stationarity, normality, dispersion ratio, zero-crossings and energy features provided statistically different unidimensional distributions for swallows and aspirations, by a rank sum test (*p *≤ 8.5 × 10^-4 ^for each of the five features). Note that stationarity, normality and dispersion ratio can be considered as capturing time domain information, whereas energy and zero-crossing features relate to spectral information in the signals. Each of the five features is described below.

#### Stationarity

Weak stationarity implies that the mean and variance of the signal do not change over time. Determination of stationarity is important in selecting the appropriate analytical method, such as in the fractal characterization of time series [37]. The reverse arrangement test is a simple, non-parametric test for stationarity [38]. For convenience, we used the associated test statistic as the stationarity feature, that is,



Here, *A *is the number of reverse arrangements in the signal, and *μ*_*A *_and *σ*_*A*_, defined as in [6], only depend on the length of the signal.

Under the null hypothesis of stationarity, *z*_*A *_is distributed as a standard normal with zero mean and unit variance. Hence, at the 5% significance level, |*z*_*A*_| < 1.96 for a stationary signal. For a step-by-step procedure for calculating the number of reverse arrangements, *A*, please see [38].

#### Normality

Normality measures the adherence of a signal's amplitude distribution to that of an ideal normal distribution. Suppose we have a signal of length *n*. To compute this feature, the signal's amplitude is first divided into a finite number of intervals or bins, *I*, *I *<<*n*, over the range of variation. We then count the number of times the signal's amplitude falls into each bin, yielding so-called observed frequencies. For each bin, we can also compute an expected frequency, that is the number of observations one would expect had the signal's amplitude been normally distributed. From these quantities, we derived a normality feature, *N*, on the basis of the Chi-square test for normality [39], namely,.



In the above, *n*_*i *_is the observed frequency in the *i*^*th *^bin, and  is the expected frequency in the same bin under the null hypothesis of a normal amplitude distribution.

#### Dispersion ratio

Dispersion ratio is the ratio between the mean absolute deviation and the interquartile range of a signal. The mean absolute deviation, *MAD*, can be found by,



where med(x) is the median of the signal. The interquartile range, denoted here as *IQR*, is defined as

*IQR *= *q*_0.75 _- *q*_0.25 _    (4)

where *q*_0.25 _and *q*_0.75 _are the first and third quartiles of the signal's amplitude distribution. The dispersion ratio is expressed as,



and can be interpreted as capturing the difference between a non-robust (mean absolute deviation) and a robust (interquartile range) estimate of spread. This feature thus roughly reflects the nature and multiplicity of atypical observations within the signal. In the absence of such a typical observations, the ratio would tend to unity. For further details about the constituent computations for this feature, please see for example [40].

#### Zero-crossings

The number of zero-crossings in a signal is an often used feature which can be easily computed in the time domain, but loosely reflects the overall frequency content of the signal. Suppose we have a signal with *n *samples, {*x*_1_,...,*x*_*n*_}. We estimated the zero-crossing feature by,

*Z *= *card*{*x*_*i *_| sign(*x*_*i*_) ≠ sign(*x*_*i*+1_)} - *card*{*x*_*j *_| sign(*x*_*j*_) = 0}     (6)

for *i *= 1,...,*n *- 1 and *j *= 1,...,*n*. In the above, *card *denotes cardinality of the set while sign(*x*) is the sign function. We subtract the actual number of points whose value is zero (the second term above) to avoid double-counting the number of zero-crossings.

#### Energy

Since pediatric aspiration signals are often non-stationary [6], we adopted a wavelet-based estimate of signal energy, previously proposed as a discriminatory feature for the classification of biomechanical signals [41,42]. In particular, the chosen energy feature was the sum of the squared detailed coefficients at the fourth level of a five-level Daubechies-4 wavelet transform [43]. This feature represents the energy of the low frequency components in the observed accelerometry signal. Given a 5-level discrete wavelet decomposition (DWT) of a signal *x*_*i *_into an approximation (*a*_5_) and detail signals (*d*_5_,...,*d*_1_), i.e.,

*DWT *[*x*_*i*_] = [*a*_5_|*d*_5_, *d*_4_, *d*_3_, *d*_2_, *d*_1_]     (7)

the selected energy feature is simply given as



where due to successive downsampling of the signal, there are *n*/16 coefficients at the 4^*th *^level of decomposition. The choice of this feature was motivated by the fact that swallowing signals tend to contain frequency peaks from a few hundred Hertz to around 1 kHz [36,44], whereas our observations suggest that aspirations signals have higher pitched components.

### Radial basis classifier design

A radial basis function network, a highly versatile and easily implementable classifier, was chosen to facilitate the selection of decisive features. The radial basis function network is a universal function approximator [45]. In other words, given sufficient training samples and unlimited hidden units, the network is able to model any continuous function between the inputs and outputs. It has also been argued that the radial basis network is suited to multimodal data [46], sports favourable convergence rates and provides statistically consistent estimation [47]. Additionally, radial basis function networks can be trained with standard linear techniques, circumventing gradient descent training issues that plague conventional back-propagation trained feedforward networks [48]. Radial basis networks have been deployed frequently in rehabilitation engineering, for example, in the control of neural prostheses [49] and in the design of an intelligent wheelchair guidance system [50].

**Figure 2:**
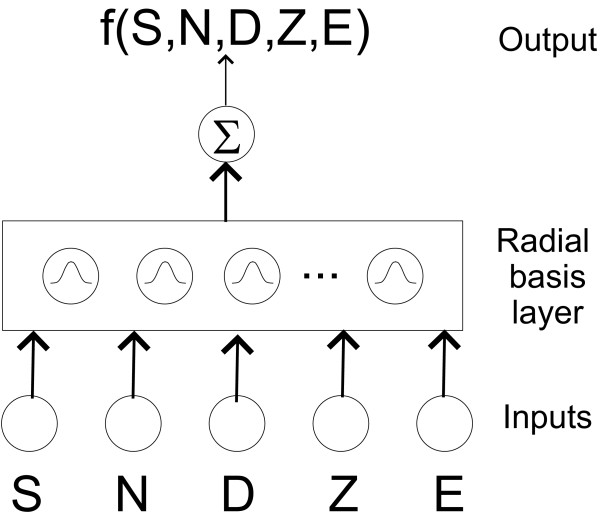
Radial basis function architecture for aspiration detection, shown here with all five features. S = stationarity, N = normality, D = dispersion ratio, Z = zero-crossings, E = energy.



For our experiments, the number of inputs to the network equaled the number of features, ranging from 1 to 5. The network had a single output, coded to represent aspirations by a numerical value of 0.9 and swallows with a value of 0.1. These values were chosen to mitigate saturation of the basis functions. The gaussian radial basis function was selected for its proven approximation capabilities. The number of radial basis units was increased as necessary during training to achieve the targeted performance. Initially, all networks started with two basis units and this was increased by five at each training iteration to a maximum equal to the number of training exemplars. The termination criterion for training was a successive error of 0.1. This coarse error margin was considered sufficient since our target values of 0.1 and 0.9 can be resolved at this precision. Figure 2 portrays the radial basis function network architecture for the five input feature case. All other networks would have a subset of the five features and hence fewer input nodes. For clarity, we have intentionally omitted bias factors at each layer and have used bold arrows to denote full connections between layers, i.e. every node is connected to every other node in the next layer. The output function can be written as a linear summation of the gaussian kernels evaluated at the current input vector, x,

where *w*_*i *_is the weight from the *i*^*th *^radial basis to the output layer, *G*(·) is the radial basis kernel, c_*i *_is the center of the *i*^*th *^radial basis function and ||·|| denotes Euclidean distance. In Figure 2, we have x = [*SNDZE*]^*T*^. For further details on radial basis network architectures and training algorithms see [45,51]. The simulation experiments were conducted in MATLAB.

### Evaluation of feature sets

To identify which combinations of the above features yield the best discriminatory potential with a radial basis classifier, we formed all possible unique combinations of one through five features. In total, there were (5, *m*) = 31 unique feature combinations, where *C*(*n*, *m*) means *n *choose *m *combinations. For each feature combination, we performed a 10-fold cross-validation [48] estimate of various classification performance measures described below. The 90%–10% split was deemed to provide a reasonably sized test set based on the sample size of available data (100 swallows + 94 aspirations = 194 instances).

The interfeature correlations were calculated to gauge the amount of overlapping information captured by each feature. Additionally, the correlations between each feature and descriptive aspiration label (Table 1), bolus consistency, participant's age and gender were computed. These correlations would hopefully help to ascertain the clinical information, if any, reflected in each feature.

### Classifier performance measures

To judge the relative merits of each feature combination, we computed some standard performance measures. Before discussing these measures, we need to clarify the meaning of some terminology in the context of the present application. Positive and negative detections refer to classification decisions of aspirations and swallows, respectively. Therefore, a false positive (FP) is the event of classifying a vibration signal as an aspiration when a swallow has actually occurred, whereas a false negative (FN) is the event of classifying a vibration signal as a swallow when an aspiration has actually occurred. Likewise an aspiration that is correctly classified as such is a true positive (TP) and a correctly classified swallow is a true negative (TN). The most common measure of classifier performance is accuracy, defined as



where the denominator is simply the total number of attempted classifications and corresponds to the size of the test set in the each cross-validation iteration. Accuracy only gives a global sense of classifier performance and may not be very meaningful when the number of swallows and aspirations in the test set are unbalanced.

We thus also examine classifier performance on aspirations and swallows individually. Sensitivity is the proportion of actual aspirations that are correctly classified as aspirations,



whereas specificity is the proportion of actual swallows that are correctly classified as swallows,



Lastly, the adjusted accuracy [52], a measure which accounts for unbalanced sample sizes of positive (aspirations) and negative (swallows) events was also computed. The adjusted accuracy, combines sensitivity and specificity into a single measure given simply by



## Results

### Sample signals

**Figure 3:**
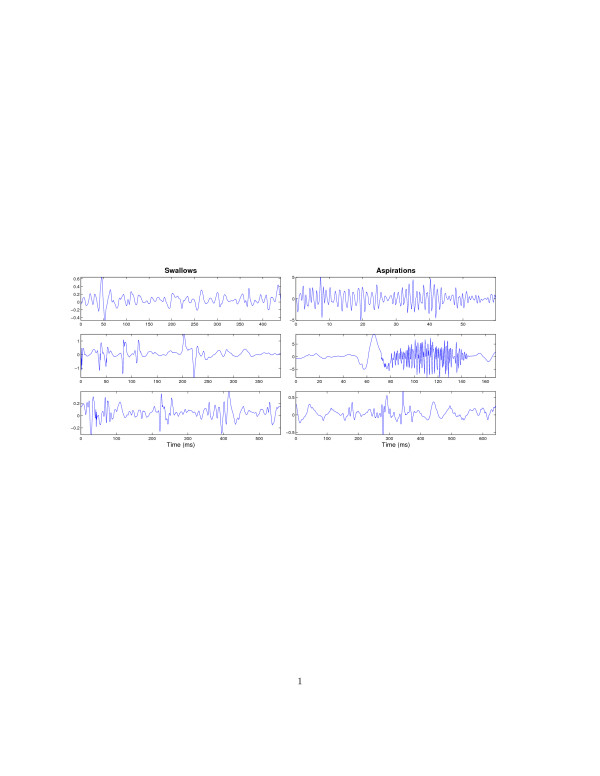
Sample swallow signals on the left and aspiration signals on the right. Note that swallows are typically longer in duration and dominated by low frequency components. Aspirations come in many flavours, some with noticeable high frequency elements (top and middle graphs on right side), but others with predominantly low frequency components (bottom right graph).

Figure 3 portrays some typical aspiration and swallow signals recorded from pediatric clients during the modified barium swallow procedure. Immediately, one notices that swallow signals are typically longer in duration and dominated by low frequency fluctuations. In contrast, aspiration signals are generally shorter, but can exhibit both remarkable high frequency components (top and middle graphs on the right hand side of Figure 3), as well as dominant low frequency trends (bottom right graph of Figure 3).

### Optimum combination of features

The classification results with the 31 unique feature combinations are tabulated in Table 2. The size of the feature set ranges from 1 to 5. The best feature combination for each size of feature set is labeled with an asterisk. Examining the adjusted accuracy column, the best two-feature combination is that of dispersion ratio and normality. This duality is slightly more sensitive but less specific than the best tripartite combination of dispersion ratio, energy and normality. However, these differences are not statistically significant (*p *> 0.2) due to the large variability in sensitivity and specificity values.

**Table 2:** Performance comparison of all possible feature combinations

**Combination**	**Accuracy**	**Sensitivity**	**Specificity**	**Adjusted Accuracy**
*D	0.711 ± 0.090	0.722 ± 0.133	0.698 ± 0.125	0.710 ± 0.089
E	0.521 ± 0.084	0.489 ± 0.170	0.589 ± 0.174	0.539 ± 0.077
Z	0.584 ± 0.115	0.703 ± 0.242	0.536 ± 0.219	0.620 ± 0.120
N	0.695 ± 0.126	0.780 ± 0.173	0.608 ± 0.165	0.694 ± 0.130
S	0.642 ± 0.099	0.557 ± 0.178	0.720 ± 0.090	0.638 ± 0.095
D-E	0.679 ± 0.101	0.656 ± 0.155	0.692 ± 0.137	0.674 ± 0.101
D-Z	0.579 ± 0.082	0.505 ± 0.195	0.673 ± 0.177	0.589 ± 0.077
*D-N	0.800 ± 0.078	0.794 ± 0.117	0.803 ± 0.128	0.798 ± 0.073
D-S	0.642 ± 0.126	0.612 ± 0.183	0.641 ± 0.219	0.627 ± 0.137
E-Z	0.563 ± 0.117	0.452 ± 0.166	0.687 ± 0.109	0.569 ± 0.118
E-N	0.758 ± 0.093	0.738 ± 0.181	0.764 ± 0.180	0.751 ± 0.090
E-S	0.537 ± 0.138	0.456 ± 0.181	0.628 ± 0.200	0.542 ± 0.141
Z-N	0.595 ± 0.134	0.226 ± 0.133	0.958 ± 0.071	0.591 ± 0.085
Z-S	0.574 ± 0.164	0.482 ± 0.304	0.693 ± 0.187	0.588 ± 0.170
N-S	0.742 ± 0.091	0.706 ± 0.146	0.783 ± 0.117	0.745 ± 0.097
D-E-Z	0.568 ± 0.128	0.481 ± 0.217	0.680 ± 0.180	0.581 ± 0.126
*D-E-N	0.821 ± 0.090	0.747 ± 0.160	0.878 ± 0.122	0.813 ± 0.085
D-E-S	0.495 ± 0.097	0.436 ± 0.194	0.532 ± 0.103	0.484 ± 0.102
D-Z-N	0.584 ± 0.139	0.304 ± 0.241	0.868 ± 0.278	0.586 ± 0.090
D-Z-S	0.605 ± 0.127	0.507 ± 0.299	0.737 ± 0.160	0.622 ± 0.143
D-N-S	0.784 ± 0.104	0.760 ± 0.176	0.809 ± 0.078	0.784 ± 0.110
E-Z-N	0.547 ± 0.109	0.071 ± 0.078	1.000 ± 0.000	0.536 ± 0.039
E-Z-S	0.553 ± 0.136	0.185 ± 0.133	0.911 ± 0.095	0.548 ± 0.083
E-N-S	0.805 ± 0.093	0.658 ± 0.168	0.922 ± 0.090	0.790 ± 0.099
Z-N-S	0.542 ± 0.127	0.072 ± 0.091	1.000 ± 0.000	0.536 ± 0.046
D-E-Z-N	0.547 ± 0.109	0.071 ± 0.078	1.000 ± 0.000	0.536 ± 0.039
D-E-Z-S	0.547 ± 0.132	0.172 ± 0.119	0.911 ± 0.095	0.542 ± 0.077
*D-E-N-S	0.811 ± 0.090	0.670 ± 0.160	0.922 ± 0.090	0.796 ± 0.095
D-Z-N-S	0.542 ± 0.127	0.072 ± 0.091	1.000 ± 0.000	0.536 ± 0.046
E-Z-N-S	0.537 ± 0.116	0.052 ± 0.081	1.000 ± 0.000	0.526 ± 0.041
*D-E-Z-N-S	0.537 ± 0.116	0.052 ± 0.081	1.000 ± 0.000	0.526 ± 0.041

Note: S = stationarity, N = normality, D = dispersion ratio, Z = zero-crossings, E = energy.

* denotes the best feature combination for each dimension of feature set.

Going from the best three to four features (dispersion ratio, energy, normality and stationarity), the classifier becomes less sensitive but more specific at identifying aspirations. Again, however the differences are not significant (*p *> 0.3).

Also noteworthy, the three-feature combination of dispersion ratio, normality, and stationarity yielded sensitivity and specificity values most comparable to the dispersion-normality duo. Both these feature combinations would be particularly amenable to implementation on a standard workhorse microcontroller as all computations can be made in the time domain, in real-time.

**Figure 4:**
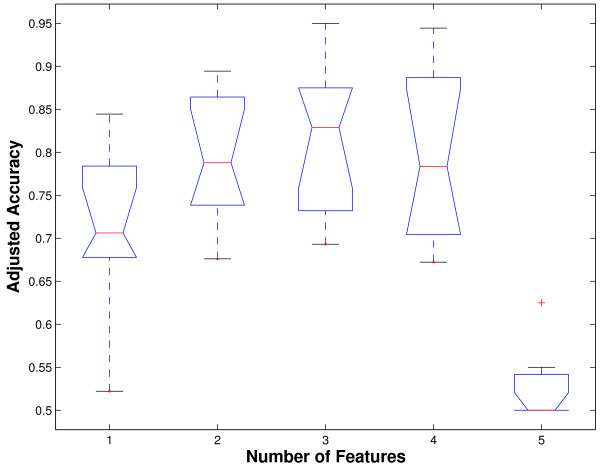
Notched boxplots showing change in adjusted accuracy as the number of features are increased from 1 to 5. Only the best feature combination for each number of features is shown.

We note that as the number of features increases, the performance improves initially, but stabilizes, then diminishes. This behavior is portrayed by the sequence of notched box plots in Figure 4. Only the cross-validated adjusted accuracies for the best feature combinations are shown. There is a statistically significant increase in adjusted accuracy from 1 to 2 features (*p *= 0.041) by the Kruskal-Wallis test. There is no significant difference (*p *= 0.9) among the accuracies using 2, 3 and 4 features. However, from 4 to 5 features, there is significant decrease in adjusted accuracy (*p *= 10^-4^). This trend is in agreement with common wisdom in pattern recognition [48]. Hence, performance is statistically equivalent with either the best 2, 3 or 4 features. From the perspective of computational economy, the fewer the features, the more desirable the solution.

### Clinical correlates

Pairwise correlation coefficients among the five features extracted from the accelerometry signals are given in Table 3. Apart from normality and zero-crossings which appear to be somewhat positively correlated, the other features are only weakly correlated. This suggests that the features are generally representing different pieces of information about the vibration signals. In conventional regression analysis, it is usually desirable to have uncorrelated independent variables [53]. The general lack of correlation implies that the selected features could also be exploited by simpler classifiers based on multivariate regression modeling.

**Table 3:** Correlation among extracted features

	**Dispersion Ratio**	**Energy**	**Normality**	**Stationarity**
**Zero-Crossings**	-0.2486	-0.1331	0.6357	-0.3888
**Stationarity**	-0.1417	-0.0690	-0.3414	
**Normality**	0.3554	-0.0829		
**Energy**	0.1881			

Pairwise correlations among the extracted features for aspirations and the four clinical variables are presented in Table 4. Surprisingly, there were no noteworthy correlations, either positive or negative. This result implies that the fundamental nature of aspiration signals, as represented by the extracted features, do not depend on bolus consistency, age and gender of the participants. Moreover, the criteria used by clinicians to assign a descriptive label to the aspiration signal are likely very different from the identified mathematical features.

**Table 4:** Correlation among features and clinical variables

	**Bolus Consistency**	**Age**	**Gender**	**Clinical Class**
**Dispersion Ratio**	-0.0098	0.0971	-0.1012	-0.0968
**Energy**	0.0190	-0.0223	0.0275	0.0300
**Normality**	0.0285	0.1064	0.0116	0.0219
**Stationarity**	-0.1808	-0.0954	-0.1019	-0.1817
**Zero-Crossings**	0.0323	-0.0027	0.0262	0.1676

## Discussion

### Features for pediatric aspiration detection

**Figure 5:**
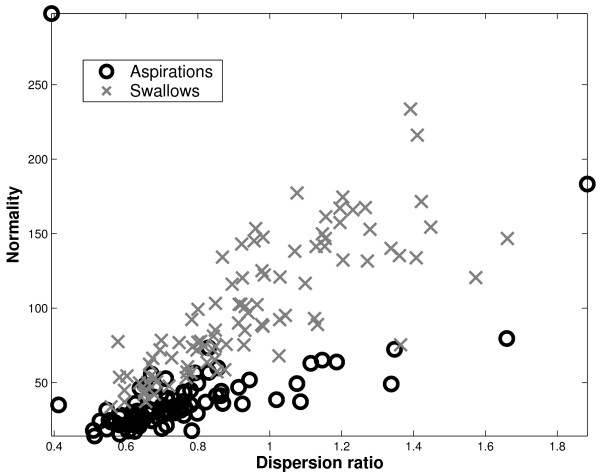
Normality-dispersion ratio plane. With these 2 features, swallows and aspirations appear to be well separated. Note that one outlying observation was omitted from this plot for the sake of clarity.

From our experiments, normality and dispersion ratio form a good feature combination in terms of separating aspirations and swallows. Figure 5 depicts the feature space for this optimal 2-dimensional feature combination. We can visually verify that swallows and aspirations are roughly quadratically separable in this feature space.

**Figure 6:**
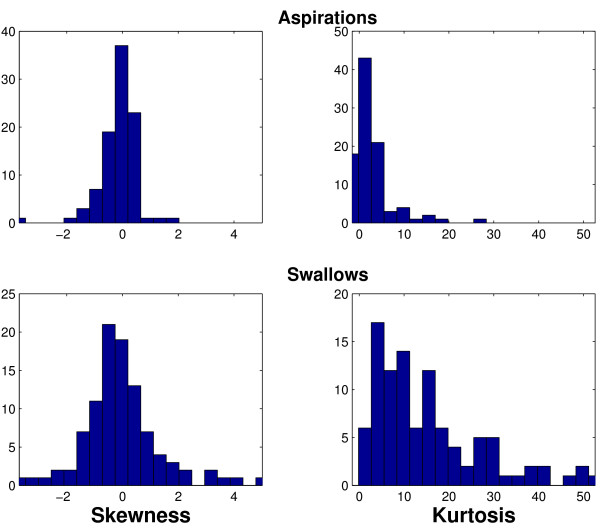
Skewness and kurtosis of aspirations (top row) and swallows (bottom row).

To understand the reason for the good separability by the normality feature, we examine the skewness and kurtosis of the empirical data. Here we use the convention that normally distributed data have 0 skewness and 0 kurtosis. Figure 6 portrays histograms of the skewness and kurtosis of aspirations in the top 2 figures and the corresponding statistics for swallows in the bottom 2 figures. While swallows have higher variability in skewness values, we see that aspirations and swallows exhibit similar skewness histograms (*p *= 0.542). These histograms suggest that amplitude distributions of both aspiration and swallow signals are generally symmetrical, although there are some positively and negatively skewed signals. Hence, the difference in normality is likely not attributable to differences in skewness.

Moving on to kurtosis, we remark that the right half of Figure 6 clearly shows that swallows are significantly more leptokurtic [38] than aspirations (*p *<< 10^-5^). This marked difference in kurtosis values is a highly probable reason for observed statistical difference in normality between aspirations and swallows. The leptokurtic nature of swallows suggests that they are more peaked than a normally distributed signal, with thicker tails. In the present application, leptokurticity may be due to the heteroscedasticity of the signals, that is, the changing variance of the signal over the course of time. Particularly, the combination of two normal signals with different variances can produce a leptokurtic signal. This kind of heteroscedastic behaviour has been identified in speech signals [54].

Examining the value of dispersion ratios in Figure 5, we note that aspirations tend to have dispersion ratios less than one. Bearing in mind the influence functions [55] for mean absolute deviation and interquartile ranges, we infer that aspiration signals generally sit in the "stable" region of the influence function, where in fact, the mean absolute value is less than the interquartile range. Practically, this means that aspiration signals have fewer atypical values, leading to a closer agreement between robust and non-robust spread estimates. On the other hand, swallows frequently have dispersion ratios in excess of 1.0, suggesting that outlying values are exerting undue influence on the non-robust mean absolute deviation value. In short, the normality and dispersion ratio features seem to capture fundamental differences between aspiration and swallow signals and hence in concert, provide a good feature space for classification.

In terms of adjusted accuracy, our present results indicate that statistically, there is no need to include a third feature, at least, none of the ones we have selected.

It is important to note here that not all features are equally implementable in hardware. For instance, the energy feature described in this paper is not easily implementable with a standard microcontroller without digital signal processing capabilities. In general, features requiring spectral analysis are more difficult to implement in hardware than those requiring strictly time-domain computations.

### Aspiration classifier

The proposed feature combinations and radial basis classifier achieved approximately 80% adjusted accuracy in classifying aspirations and swallows. This accuracy level already exceeds that achievable by the best trained clinician using cervical auscultation at the bedside, where one typically achieves no better than 40 to 60% accuracy [22,24]. Recently, in a study involving eleven expert judges and a small sample of 20 stethoscopic sounds of "normal" and "abnormal" swallowing, individual rater specificity and sensitivity for aspiration/penetration detection were only 66% and 62%, respectively [30]. We thus argue that the proposed classifier is an important first step towards developing a non-invasive aspiration detection method in the paediatric population.

A classifier can make false positive and false negative errors, each with a potentially different associated cost. From the medical perspective, clearly missing multiple aspirations (false negatives) is a costly error bearing serious health consequences described previously. However, from a caregiver perspective, rampant false alarms may unnecessarily limit oral feeding, which in turn may have negative nutritional impact. In developing a clinically useful system, the tradeoff between these two errors should be carefully considered and perhaps tailored to the individual client and family situation.

While we have elected to use a universal function approximator in the radial basis function network, knowing some discriminatory features, one could certainly consider simpler alternatives such as a piecewise linear discriminant classifier [48] or a nearest-neighbour algorithm [56] as a viable and perhaps more suitable solution for micro-controller implementation.

### Hardware implementation of the aspirometer

We contend that the proposed classifier can be easily realized in hardware as a portable and non-invasive swallow-safety monitor. In this section, we briefly describe one such implementation, noting that many other variations are possible. We have coined the term "aspirometer" for the the hardware device that encapsulates the proposed classification algorithm. A working prototype of this aspirometer has been constructed at Bloorview Kids Rehab in Toronto, Canada.

**Figure 7:**
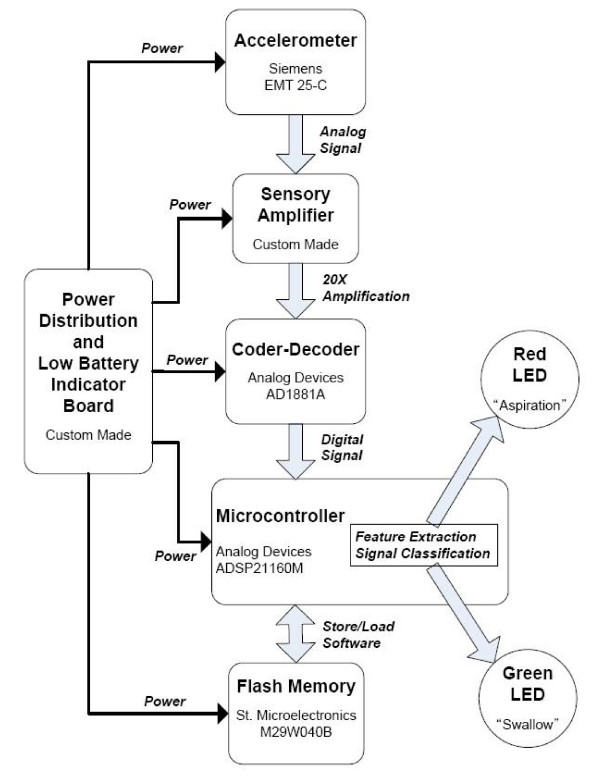
Overview of the aspirometer: one possible implementation of the proposed classifier.

Figure 7 is a block diagram of the aspirometer. The prototype consists of a single-axis accelerometer (EMT-25C, Siemens), a custom sensory amplifier, a hardware codec (AD1881A, Analog Devices), a microcontroller (ADSP 21160M, Analog Devices), a flash memory (M29W040B, St. Microelectronics), a custom power distribution and low battery indicator board and two LEDs (red and green) for visual output. The accelerometer is attached to the child's neck by way of double-sided tape. The amplifier provides a twenty-times amplification with an anti-aliasing filter. The microcontroller performs data acquisition, feature extraction, classificaiton and output LED control. The microcontroller's memory is volatile, hence the software is stored in the flash memory and retrieved by the microcontroller each time the device is initialized. The red and green LEDs indicate aspiration and swallow, respectively. The entire unit is powered by two high energy nickel metal hydride (NiMh) batteries (2700 mAh, 1.2 V, Sanyo).

### Rehabilitative strategies

Upon aspiration notification by the aspirometer, the caregiver may intervene in a variety of different ways, in accordance with recommendations by the clinical care team. For example, the caregiver might encourage the child to attempt a voluntary cough to bring up any residue that may have entered the airway. For subsequent feeding, the bolus consistency may be appropriately modified or the the position of the child may be adjusted [21]. The caregiver may also reduce the speed and/or volume of presentation of food to facilitate subsequent swallows. Recurrent aspiration warnings, especially in combination with clinical evidence such as chest disease particularly if recurrent, evidence of aspiration on a chest X-Ray, recurrent fevers, unexplained choking with feeds, coughing with feeds, a raspy breathing pattern, wet voice or deteriorating breathing pattern while feeding would indicate the need for videofluoroscopic re-assessment by the clinical care team.

### Potential impact of an aspirometer

It is anticipated that an aspirometer device would have significant impact in pediatric rehabilitation, primary and tertiary care, particularly in individuals who tend to aspirate silently. Firstly, reliable, non-invasive aspiration detection would be available at bedside, at home, at school and in the community. Neither clinical experts nor expensive equipment would be required. Caregivers would have a peace of mind when feeding the child with dysphagia. Secondly, an aspirometer device could potentially facilitate a better referral strategy for videofluoroscopic examinations (VFE). Currently, in many remote or medically under-serviced communities, radiology suites are in short supply and waiting lists can be many months long. The aspirometer might serve as a pre-screening tool to identify those for whom VFE is warranted. Waiting times for videofluoroscopy could conceivably be reduced as a result.

### Limitations and future extensions

The current classifier formulates its decision solely on a unidimensional vibration signal and has no knowledge of other physiological indications, such as discoordinated breathing, which may accompany aspiration. Hence, the addition of other physiological data such as ventilation, facial muscle activities, and heart rate may further enhance and temporally advance aspiration detection. However, it would be technically challenging to integrate these different information sources into a portable and self-contained device.

The current classifier was constructed from 94 aspiration and 100 swallow samples. This sample limits the dimensionality of the feature space in which class densities may be estimated, as per the curse of dimensionality [57]. The sample size also constrains the number of folds used in cross-validation. With larger samples, we may be able to minimize the variance in the estimated performance indices. We note however, that as noted in [6], it is extremely difficult to assemble a large database of pediatric aspiration signals due to their relatively infrequent occurrence in a clinical setting.

The current results have been obtained only with pediatric data and can not be generalized to adults. Future studies employing similar methodology with adults are required to ascertain the generalizability of automatic aspiration detection using the proposed features and classifier.

We have discussed five candidate features in this paper. Further research into discriminatory features may enhance the dispersion-normality duality to provide even higher specificity and sensitivity. Further, the generalization of the swallow class to a generic non-aspiration class which includes guttural sounds, vocalizations, coughing and crying noises may help to reduce false positives.

## Conclusion

The proposed pediatric aspiration classifier provides promising accuracies. It is particularly conducive to implementation as a portable, non-invasive "aspirometer" device. Dispersion ratio and normality prove to be especially good features for distinguishing aspirations from safe swallows, while sub-band energy appears to be a useful additional feature. A radial basis network offers a versatile architecture for classifier exploration but simpler classifiers may also be suitable on the basis of the proposed feature spaces. The proposed classifier can be further enhanced by considering other features and expanding the scope of swallowing events for training. The ultimate application of such a classifier might be a wearable detection/intervention system for the management of aspiration risk.

## Authors' contributions

JL wrote the abstract, methods, results and discussion sections of the manuscript. JL also generated the tables. SB designed the hardware implementation of the classifier algorithm, tested the microcontroller implementation and contributed to the hardware section of the paper. She also characterized the accelerometer employed in the study. MC spearheaded the set-up of the instrumentation and reviewed videofluoroscopic data. DK reviewed videofluoroscopic data and advised throughout the study. GB recruited clients for the study, identified aspiration events during videofluoroscopy, reviewed videofluoroscopic data and contributed to parts of the discussion section. MC, DK and GB initiated and completed pilot studies. TC oversaw data collection and data analysis, wrote the background, data collection and selected portions of the methods, results and discussion sections of the manuscript. He also generated the graphs in the results section.
